# Is Development of High-Grade Gliomas Sulfur-Dependent?

**DOI:** 10.3390/molecules191221350

**Published:** 2014-12-19

**Authors:** Maria Wróbel, Jerzy Czubak, Patrycja Bronowicka-Adamska, Halina Jurkowska, Dariusz Adamek, Bolesław Papla

**Affiliations:** 1 Chair of Medical Biochemistry, Jagiellonian University Medical College, Kopernika 7 St., Kraków 31-034, Poland; E-Mails: jerzy.czubak@uj.edu.pl (J.C.); mbbronow@cyf-kr.edu.pl (P.B.-A.); mbjurkow@cyf-kr.edu.pl (H.J.); 2 Department of Pathomorphology, Jagiellonian University Medical College, Grzegórzecka 16 St., Kraków 31-531, Poland; E-Mails: dariusz.adamek@uj.edu.pl (D.A.); bolekpapla@gmail.com (B.P.)

**Keywords:** cysteine, γ-cystathionase, glioma, human brain, hydrogen sulfide, 3-mercaptopyruvate sulfurtransferase, rhodanese, sulfane sulfur

## Abstract

We characterized γ-cystathionase, rhodanese and 3-mercaptopyruvate sulfurtransferase activities in various regions of human brain (the cortex, thalamus, hypothalamus, hippocampus, cerebellum and subcortical nuclei) and human gliomas with II to IV grade of malignancy (according to the WHO classification). The human brain regions, as compared to human liver, showed low γ-cystathionase activity. The activity of rhodanese was also much lower and it did not vary significantly between the investigated brain regions. The activity of 3-mercaptopyruvate sulfurtransferase was the highest in the thalamus, hypothalamus and subcortical nuclei and essentially the same level of sulfane sulfur was found in all the investigated brain regions. The investigations demonstrated that the level of sulfane sulfur in gliomas with the highest grades was high in comparison to various human brain regions, and was correlated with a decreased activity of γ-cystathionase, 3-mercaptopyruvate sulfurtransferase and rhodanese. This can suggest sulfane sulfur accumulation and points to its importance for malignant cell proliferation and tumor growth. In gliomas with the highest grades of malignancy, despite decreased levels of total free cysteine and total free glutathione, a high ratio of GSH/GSSG was maintained, which is important for the process of malignant cells proliferation. A high level of sulfane sulfur and high GSH/GSSG ratio could result in the elevated hydrogen sulfide levels. Because of the disappearance of γ-cystathionase activity in high-grade gliomas, it seems to be possible that 3-mercaptopyruvate sulfurtransferase could participate in hydrogen sulfide production. The results confirm sulfur dependence of malignant brain tumors.

## 1. Introduction

l-Cysteine desulfuration, a source of sulfane sulfur-containing compounds, has not been extensively investigated in the human brain. Sulfane sulfur is involved in the detoxification of cyanide and inorganic sulfide, the incorporation of sulfur in iron-sulfur centers of redox proteins, enzyme activity regulation through a mechanism that involves the incorporation of sulfur, sulfuration of tRNA; it also has an antioxidant potential and may affect the toxic function of exogenous xenobiotics or drugs (not reviewed here, but see [1,2]). Sulfane sulfur atoms are easily ejected as elemental sulfur, transferred to another sulfur atom or reduced to H_2_S by thiols [1,3,4,5].

**Scheme 1:**
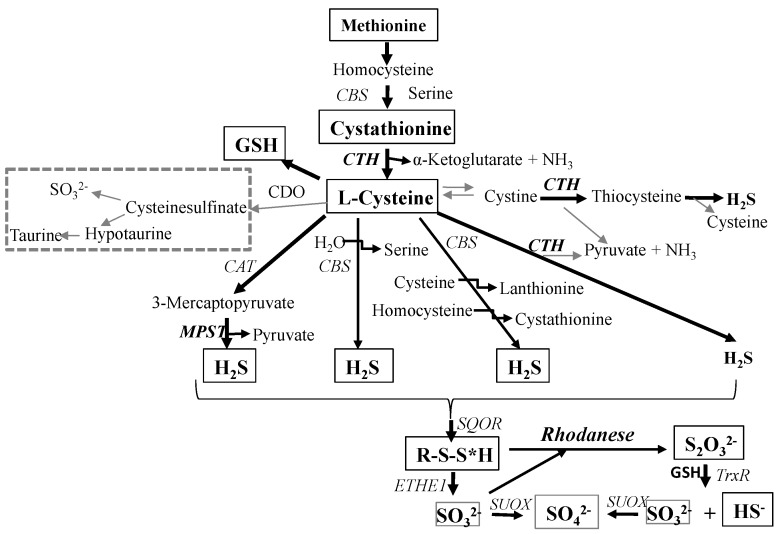
l-Cysteine desulfuration pathways.

The objective of the present study was to investigate l-cysteine desulfuration (Scheme 1) in various regions of human brain and in human gliomas of various grades of malignancy, through the estimation of the activity of enzymes participating in the formation and metabolism of sulfane sulfur compounds, *i.e.*, cystathionine γ-lyase (CTH, EC 4.4.1.1), 3-mercaptopyruvate sulfurtransferase (MPST, EC 2.8.1.2), rhodanese (thiosulfate sulfurtransferase, EC 2.8.1.1), and through the determination of the level of sulfane sulfur, cysteine, glutathione and cystathionine.

*CBS* (Cystathionine β-synthase), *CTH* (Cystathionine γ-lyase), *MPST* (3-Mercaptopyruvate sulfurtransferase), *CAT* (Cysteine aminotransferase), *CDO* (Cysteine dioxygenase), *SQOR* (Sulfide:quinone oxidoreductase), *ETHE1* (Persulfide dioxygenase), *SUOX* (Sulfite oxidase), TrxR (Thioredoxin reductase). Based on Jurkowska *et al.* [6].

Human gliomas are the most common primary central nervous system neoplasms. Like many human cancer cell lines (breast, lung, colon, kidney, bladder, melanoma, glioblastoma, *etc.*) and primary tumors, gliomas have absolute requirements for methionine [7,8]. Methionine, an essential amino acid, participates in protein synthesis, is required for the formation of the polyamines spermine and spermidine, is the major source of methyl groups for methylation of DNA and other molecules, and serves as a precursor of cysteine and glutathione. The biochemical mechanism for methionine dependency has been studied extensively, but the fundamental mechanism remains unclear. Tumor cells cultured in the absence of methionine are known to have a reduced transmethylation capacity. Methionine depletion induces a decrease in glutathione levels and some studies indicate that antitumor activity of methionine and homocysteine-free diet is better than that of a methionine-deficient, homocysteine-containing diet [9].

We have previously described the sulfane sulfur dependency of astrocytoma U373 cells [10,11] and prostate cancer PC-3 cells [12] proliferation. The inhibition of the proliferation was accompanied by an increase in the level of sulfane sulfur and in the expression and activity of MPST and γ-cystathionase. An opposite effect was observed for mouse astrocytes—a decreased cell proliferation accompanied by a decreased MPST activity was correlated with a decreased sulfane sulfur level [10]. This metabolic difference opens interesting perspectives for nutritional strategies in anticancer therapy.

## 2. Results and Discussion

Despite the rising interest in H_2_S biochemistry, fundamental questions remain regarding H_2_S formation, conversions and regulation of these processes in human brain. H_2_S biogenesis is the apparent by product of three enzymes, MPST, cystathionine β-synthase (CBS) and CTH. Hence, different pathways for H_2_S generation might be operational in different cell types reflecting the tissue distribution of the individual enzymes and under normal conditions *versus* cancerous conditions.

### 2.1. MPST, Rhodanese and γ-Cystathionase Activities and the Level of Sulfane Sulfur in Human Brain Regions

In a comparison of the values of the specific activity of MPST, rhodanese and CST in all the investigated brain regions, MPST was demonstrated to show the highest specific activity. The highest value of the activity of MPST was found in the thalamus and it was significantly higher than the value determined in the hypothalamus (Table 1). There were no significant differences between the values determined in the other investigated human brain regions and they oscillated between 600–700 nmol·mg^−1^·min^−1^. All the values of MPST activity determined in the brain regions were at least 10 times lower than the value determined in human liver homogenates. Similarly, rhodanese activity was about 15 times lower in human brain regions as compared to human liver and its value oscillated between 139 ± 50 nmol·mg^−1^·min^−1^ in the parietal cortex and 222 ± 63 nmol·mg^−1^·min^−1^ in the thalamus, with no statistically confirmed differences between the two regions. CTH has very low specific activity in the brain; nevertheless, CTH activity in human brain is still many times higher than that observed in mouse brain—the presence of CTH transcript was confirmed predominantly in neuronal cells in the human brain regions, indicating the potential role of the enzyme in the synthesis of cysteine and/or H_2_S in neuronal cells [13]. The difference between CTH activity in human brain regions and human liver was not as huge as in case of MPST or rhodanese. It was only about 2–3 times higher in the liver. The value of CTH activity was the highest in the cerebellum, hypothalamus, parietal cortex, and the lowest in the thalamus and hippocampus. The value in the thalamus was significantly lower in comparison to the cerebellum, hypothalamus and parietal cortex. The level of sulfane sulfur was less than two times lower in comparison to the value determined in liver homogenates. Essentially the same level of sulfane sulfur was found in all the investigated brain regions (Table 1). This may suggest sulfane sulfur homeostasis that can be important for function of some proteins/enzymes [1,14]. However, the difference of the level of sulfane sulfur between the thalamus and hippocampus was confirmed by statistical analyses.

**Table 1:** The activity of MPST, rhodanese and γ-cystathionase and the level of sulfane sulfur in various regions of human brain.

Brain Regions	MPST	Rhodanese	CTH **	Sulfane Sulfur
	nmol·mg^−1^·min^−1^			nmol·mg^−1^
Cerebellum	639 ± 103	212 ± 44	0.7 ± 0.2 *	191 ± 41
Hypothalamus	745 ± 119	200 ± 54	0.7 ± 0.3 *	221 ± 53
Thalamus	841 ± 142 *	222 ± 63	0.4 ± 0.1 *	209 ± 44 *
Nuclei subcortical	732 ± 170	179 ± 68	0.6 ± 0.4	197 ± 40
Hippocampus	671 ± 205	177 ± 67	0.4 ± 0.3	225 ± 63
Frontal cortex	627 ± 181	144 ± 35	0.5 ± 0.4	231 ± 49
Parietal cortex	574 ± 142	139 ± 50	0.7 ± 0.3 *	228 ± 70
Liver	7224 ± 2782	3031 ± 1128	1.2 ± 0.8	393 ± 133

Mann-Whitney * *p* < 0.05 for: MPST Thalamus *vs.* Hypothalamus, CTH: Thalamus *vs.* Cerebellum, Hypothalamus, Parietal cortex, Sulfane sulfur: Thalamus *vs.* Hippocampus; ** Data originating from [15].

### 2.2. MPST, Rhodanese and Cystathionase Activities and Sulfane Sulfur Level in Human Brain Gliomas

**Figure 1:**
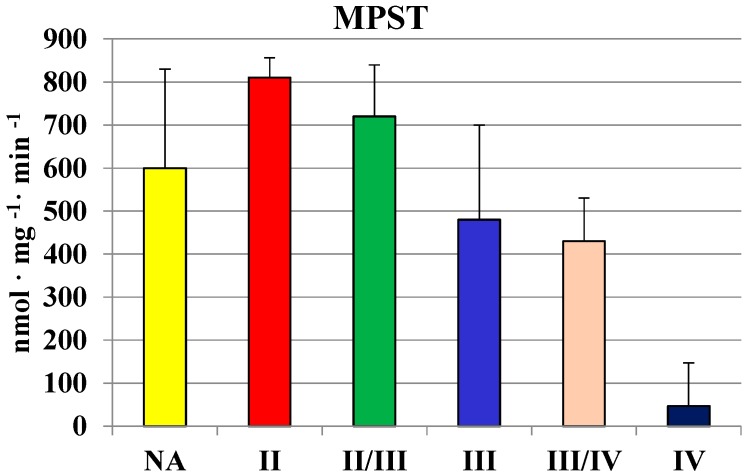
The activity of MPST in human brain gliomas of various grades of malignancy.

The activity of MPST was investigated in homogenates of gliomas of various grades of malignancy. The activity seemed to decrease along with the grade of malignancy (Figure 1).

**Figure 2:**
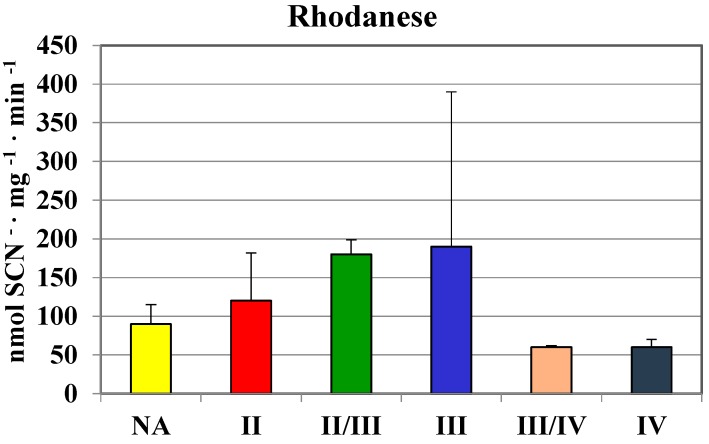
The activity of rhodanese in human brain gliomas of various grades of malignancy.

**Figure 3:**
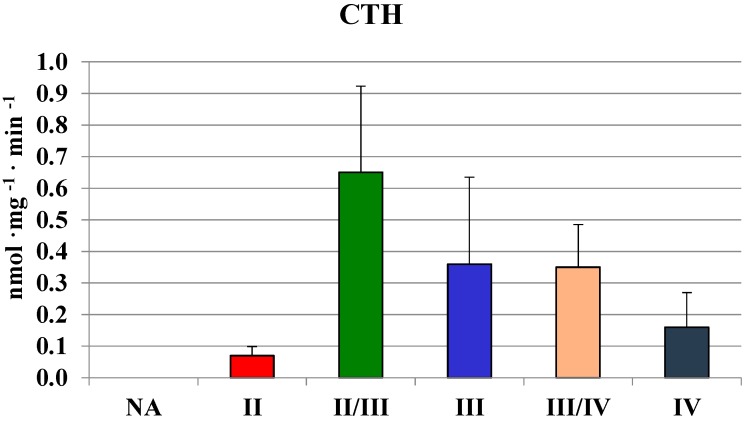
The activity of CTH in human brain gliomas of various grades of malignancy.

The highest values in homogenates of gliomas with the second and II/III grade of malignancy were comparable to those found in human brain regions (Table 1). In gliomas with the fourth grade of malignancy, the activity of MPST was absent. Similarly, gliomas with the III/IV and IV grade of malignancy showed a much lower rhodanese (Figure 2) and CTH activity (Figure 3) than the lower grade gliomas. Interestingly, gliomas with an average grade of malignancy showed the highest values of MPST, rhodanese and CTH activity.

The value for NA was not presented because slices were not sufficiently large to determine it along with all other determinations.

**Figure 4:**
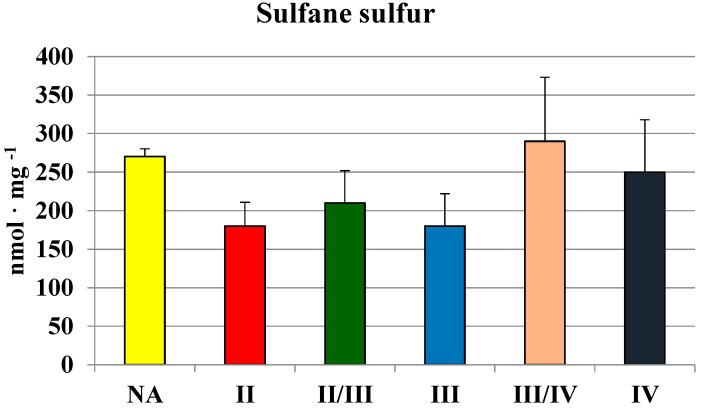
The level of sulfane sulfur in human brain gliomas of various grades of malignancy.

Although MPST and CTH activities were the lowest in glioma IV homogenate, the level of sulfane sulfur was not (Figure 4). There were no significant differences between sulfane sulfur content in homogenates of gliomas with different grade of malignancy—the level was rather stable and it seemed to be even higher in III/IV and IV grades. These observations became more apparent when we compared the mean values of MPST, rhodanese and cystathionase activity, and sulfane sulfur level in human brain and gliomas (Table 2). Despite lower MPST and CTH activity, two sulfane sulfur-generating enzymes, its level remained high.

Table 2 presents the mean value of MPST, rhodanese and cystathionase activity, and sulfane sulfur level in human brain and gliomas. The average CTH and rhodanese activities in gliomas were more than two times lower in comparison to the average values in human brain. However, the level of sulfane sulfur was not diminished; malignant cells with multiple defects in sulfur metabolism are known to require sulfane sulfur for proliferation *in vitro* [10,16,17]. Increasing evidence suggests that the mechanism of anticancer action of garlic compounds, precursors of sulfane sulfur, may involve modulation of signal transduction pathways [18,19].

**Table 2:** The mean value of MPST, rhodanese and cystathionase activity, and sulfane sulfur level in human brain and gliomas.

Tissues	MPST	Rhodanese	CTH	Sulfane Sulfur
	nmol·mg^−1^·min^−1^			nmol·mg^−1^
Brain	689 ± 151	146 ± 54	559 ± 356	214 ± 51
Glioma	442 ± 118	63 ± 28	210 ± 200	250 ± 90

Values determined in all brain homogenates and values determined in all glioma homogenates were combined to calculate the mean value.

### 2.3. Cysteine, Glutathione and Cystathionine Levels in Human Brain Gliomas

**Figure 5:**
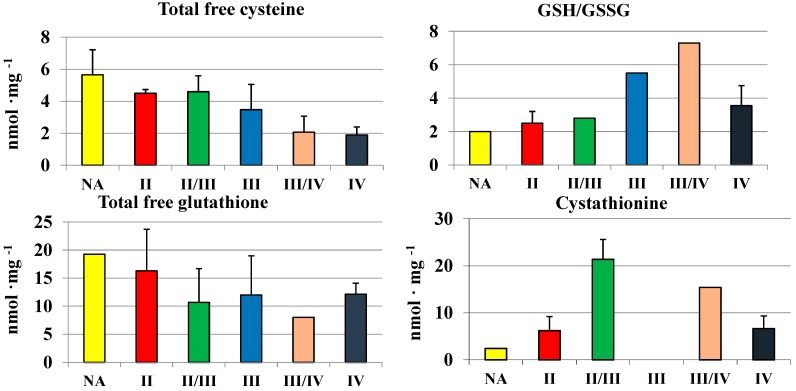
The level of total free cysteine, total free glutathione, cystathionine, and the ratio of GSH/GSSG in human gliomas.

We used the RP-HPLC method described by Bronowicka-Adamska *et al.* [15] for a simultaneous separation and quantitation of the dinitrophenyl derivative of cysteine and cystine, reduced (GSH) and oxidized glutathione (GSSG), and cystathionine in brain samples. The highest concentration of cysteine was noted in the thalamus, hypothalamus and subcortical nuclei. The highest level of cysteine in the thalamus corresponded with the highest level of GSH and the highest ratio of GSH to GSSG. GSH, as a reductant, plays a fundamental role in the detoxification of reactive oxygen species, which is critical to the normal function of the central nervous system and its altered levels have been reported in several pathologies, such as cancer [20,21,22,23]. Simultaneous determinations of GSH and GSSG levels in homogenates of gliomas allowed for determining the GSH/GSSG ratio, which reflects tissue redox status. Despite a slightly decreased level of total free cysteine (Figure 3) and glutathione, a high ratio of GSH/GSSG was maintained in gliomas with the highest grades, which can be important for the process of malignant cells proliferation. A changed profile of CTH activity (Figure 3) and cystathionine levels (Figure 5) was correlated—both of them decreased with the increase of the grade of gliomas malignancy. This suggests a decreased activity of cystathionine-β-synthase, generating cystathionine. In general, an average cystathionine level in higher grade gliomas (II/III, III/IV and IV) seemed to be higher in comparison to low grade gliomas (II).

Data are expressed as the mean values ± S.D. (or as the mean value only if the number of samples was not sufficient). Empty sites mean that not enough tissue was available to perform all the determinations.

Some authors stated previously [15] that cystathionine levels varied greatly between particular human brain regions—the level in the human thalamus (55 ± 15 nmol/mg) was about 11 times higher than in the cerebellum (4.7 ± 2.3 nmol/mg). It should be noted that the lowest activity of CTH detected in the thalamus (Table 1) is correlated with the highest level of cystathionine. Similarly, the highest activity of CTH detected in the cerebellum (Table 1) is correlated with the lowest level of cystathionine.

Numerous studies focused on the potential antitumor activity of methionine restriction that exploits metabolic differences between neoplastic and normal cells [8]. In patients with brain tumors, the evaluation of tumor size is based on the differential incorporation of methionine, higher within the tumor than in the surrounding healthy tissues [24]. The application of the micro-XANES technique allowed finding high accumulation of reduced sulfur (S^2−^) in cancer cells of malignant gliomas. The results indicated higher accumulation of this form of sulfur in glioma of IV grade of malignancy in comparison with the samples of II grade neoplasms [25]. The results presented in this paper allow for confirming desirability of dietary methionine restriction in the treatment of gliomas and suggest further investigations aiming at confirming whether availability of other than methionine precursors of sulfane sulfur would affect inhibition of proliferation of gliomas, which—as it has been demonstrated in the present paper—accumulate sulfane sulfur.

## 3. Experimental Section

### 3.1. Chemicals

l-Glutathione reduced (GSH), glutathione oxidized form (GSSG), l-cysteine, l-cystine, cystathionine (CTN), DL-homoserine (HSer), 1-fluoro-2,4-dinitrobenzene (DNFB), bathophenanthroline-disulfonic acid disodium salt (BPDS), acetonitrile, pyridoxal phosphate (PLP), β-nicotinamide adenine dinucleotide reduced disodium salt hydrate (NADH), l-lactic dehydrogenase (LDH), dl-propargylglycine (PPG), pyridoxal phosphate (PLP), dithiothreitol (DTT), *N*-ethylmaleimide, sodium carbonate, Folin-Ciocalteau reagent, sodium 3-mercaptopyruvate, potassium hydroxide, sodium chloride, ferric nitrate nonahydrate, sodium thiosulfate pentahydrate, sodium sulfide, PTFE filter were obtained from Sigma Chemical Co. (St. Louis, MO, USA). Trifluoroacetic acid (TFA), 2-mercaptoethanol and EDTA-Na_2_·2H_2_O were purchased from Fluka Chemie GmbH (Buchs, Switzerland). Potassium hydrogen carbonate, potassium hydroxide, copper (II) sulfate pentahydrate, potassium sodium tartrate tetrahydrate, sodium dihydrogen phosphate dihydrate, nitric acid (V), potassium dihydrogen phosphate, ammonia solution, sodium hydroxide, ethanol and 70% perchloric acid (PCA) were from Polish Chemical Reagents SA (Gliwice, Poland). Potassium cyanide were obtained from Merck (Darmstadt, Germany) and *N*-methyl-l-lysine from Bachem (Bubendorf, Switzerland). All the chemicals and HPLC solvents were gradient grade. Water was deionized by passing through an EASY pure RF compact ultrapure water system.

### 3.2. Human Tissues

#### 3.2.1. Sections of Normal Human Brain Obtained Postmortem

*Post mortem* brain and liver sections collected during autopsies performed at the Department of Pathomorphology and Department of Forensic Medicine, Jagiellonian University Medical College, Cracow, Poland, were used in this experiment if the examination performed by the attending pathologist within 12–24 h of death confirmed that they were macroscopically unchanged (conditions accepted by scientific research [26,27,28,29]. Sections of human brain (1–2 g) of the following regions: the hypothalamus (Hypothalamus), thalamus (Thalamus), hippocampus (Hippocampus), frontal cortex (Frontal cortex), parietal cortex (Parietal cortex), cerebellum (Cerebellum), subcortical nuclei (Nuclei basales) were collected from patients between 20 and 60 years of age. All the sections were isolated, placed in liquid nitrogen and stored at −76 °C until used in biochemical experiments. The experimental protocol (number KBET/199/B/2000) was approved by the Bioethical Commission, Jagiellonian University Medical College.

#### 3.2.2. Sections of Gliomas Obtained Intraoperatively

Sections of gliomas characterized by various grades of malignancy according to the World Health Organization (WHO) originating from patients between 20 and 60 years of age were collected intraoperatively at the Department of Neurosurgery in cooperation with the Department of Neuropathology, Institute of Neurology, Jagiellonian University (Table 3). All the sections of normal human brains were isolated, placed in liquid nitrogen and stored at −76 °C until used in biochemical experiments.

**Table 3:** The tissue sections of human brain gliomas with different grades of glioma malignancy.

No.	Tissue	WHO Grade	Number of Sections
1	Gliosis (around hemangioblastoma)	NA	1 section
2	Diffuse astrocytoma (fibrillary)	WHO II	1 section
3	Diffuse astrocytoma (gemistocytic)	WHO II	1 section
4	Diffuse astrocytoma (fibrillary)	WHO II	1 section
5	Diffuse astrocytoma	WHO II	1 section
6	Oligodendroglioma	WHO II	1 section
7	Oligoastrocytoma	WHO II/III	1 section
8	Diffuse astrocytoma	WHO II/III	1 section
9	Anaplastic oligodendroglioma	WHO III	1 section
10	Anaplastic astrocytoma	WHO III	2 section
11	Anaplastic astrocytoma/Glioblastoma	WHO III/IV	4 section
12	Glioblastoma	WHO IV	5 section

NA = not applicable; gliosis zone (non-neoplastic) surrounding a hemangioblastoma—WHO does determine the grade in this case.

### 3.3. Tissue Homogenates

For determinations of enzymes activity and level of sulfane sulfur, the sections of normal human brain and gliomas were weighed and homogenized in ice-cold 0.1 M phosphate buffer pH 7.5 (1 g/5 mL) for 1 min at 8000–9500 rpm using a blender homogenizer. The homogenates were centrifuged at 1600 *g* for 10 min. After centrifugation, the supernatants were used for the determination of the enzymes activity (CTH, MPST, rhodanese) and the level of sulfane sulfur and protein content.

For the RP-HPLC method, the tissues were weighed and homogenized at 8000–9500 rpm in ice-cold 10% PCA/1 mM BPDS (1 g/3 mL) (1 g tissue/3 mL solution). The homogenates were centrifuged for 10 min at 4 °C at 1400 *g*. The supernatants were used for assays immediately or stored at −80 °C until HPLC analysis. The tissues were homogenized using an Ultra-Turrax T 25 (Janke & Kunkel IKA-Labortechnik Company, Staufen, Germany). The homogenates were centrifuged using a MPW 375 centrifuge (MPW MED Instruments, Warszawa, Poland) or Hettich Universal 16 centrifuge (Hettich AG, Kloten, Switzerland).

### 3.4. Enzyme Assay

The MPST activity was assayed according to the method of [30] following a procedure described in our earlier paper [31]. The enzyme activity was expressed as nmoles of pyruvate produced during 1 min incubation at 37 °C per 1 mg of protein. The rhodanese activity was assayed by Sőrbo’s method, following a procedure described in [31]. The enzyme activity was expressed as nmoles of SCN^−^ formed during 1 min incubation at 20 °C per 1 mg of protein. The γ-cystathionase activity (CTH) was determined according to Matsuo and Greenberg [32] with the modification described by Czubak *et al.* [33]. The activity of cystathionine was expressed as nmoles of 2-ketobutyrate formed during 1 min incubation at 37 °C per 1 mg of protein.

### 3.5. Sulfane Sulfur and Protein

Sulfane sulfur was assayed by the method of Wood [34], based on cold cyanolysis and colorimetric detection of ferric thiocyanate complex ion. Protein was determined by the method of Lowry [35] using crystalline bovine serum albumin as a standard.

### 3.6. RP-HPLC (Reverse Phase High Performance Liquid Chromatography)

The RP-HPLC method of Dominick *et al.* [36] with modifications [15,37] was used to determine the levels of such metabolites as cysteine, cystine, cystathionine, reduced (GSH) and oxidized glutathione (GSSG) in incubation mixtures. The samples were separated on a 4.6 mm × 250 mm Luna C_18_ (5 µm) column with a Phenomenex Security Guard column filled with the same packing material. The chromatographic system consisted of LC-10 Atvp Shimadzu pumps, four channel degassers, a column oven and a Shimadzu SIL-10 Advp autosampler. Chromatographic peaks were measured by a Shimadzu SPD-M10Avp-diode array detector. A mobile phase consisting of solvent A (water/0.1% TFA) and solvent B (acetonitrile/0.1% TFA) was used to elute the samples. The samples were eluted with 20% B after injection, followed with a 35-min linear gradient to 55% B and 10 min isocratic period at 55% B, then a 15-min linear gradient to 100% B and 10-min isocratic period. The column was then re-equilibrated to the initial conditions for 15 min. All the HPLC solvents were HPLC gradient grade. The samples were filtered through a 0.20 µm PTFE filter. Analyses of 20 µL of the samples were performed at a flow rate of 1.0 mL/min at 20 °C with diode array detection at 365 nm.

#### 3.6.1. Standard Curves

Stock solutions were prepared for standard curves as follows: 2.4 µM *N*^ε^-methyllysine, 1.2 µM l-cysteine, 1.2 µM l-cystine, 1.2 µM GSH, 1.2 µM GSSG, and 2,2 µM cystathionine. All the stock solutions were prepared in 10% PCA/1 mM BPDS except for *N*^ε^-methyllysine, which was prepared in water. Standard curves were generated in the supernatant obtained from the cells in the range from 13 to 75 nmol of each compound per mL.

#### 3.6.2. Sample Preparation

The incubation mixture was prepared by taking 100 μL supernatant, 20 μL *N*^ε^-methyl-l-lysine (2.4 µM solution in proportion 1:10), 40 μL 10% PCA/1 mM BPDS, 96 μL 2 M KOH-2.4 M KHCO_3_, 200 μL 1% DNFB. The sample was derivatized overnight at room temperature in the dark. Prior to injection, the sample was acidified with 30 μL 70% PCA and clarified by centrifugation at 5600 *g* for 2 min.

### 3.7. Statistical Analysis

All the data of the RT-HPLC experiments represent the average of three to seven determinations. Values in Tables were summarized as mean ± standard deviation of the mean. The significance of the differences between mean values was calculated using the Student’s *t* Test. Statistical analysis of differences between brain regions was performed using the Mann-Whitney test.

## 4. Conclusions

Since biochemical changes may be related to the growth rate of cancer cells, they can be thought of as markers of tumor cell proliferation. Elevated levels of cystathionine are found in urine of patients with neuroblastoma due to the specific block in transsulfuration resulting from the absence of CST in the malignant tissue [38]. Human gliomas with high grade of malignancy are characterized by a relatively high level of sulfane sulfur and a lower activity of MPST, rhodanese and CTH in comparison to normal brain regions, high levels of cystathionine and high ratios of GSH/GSSG in comparison to gliomas with low grade of malignancy.

## Abbreviation

CTH: γ-cystathionase
GSH: glutathione, reduced form
GSSG: glutathione, oxidized form
MPST: 3-mercaptopyruvate sulfurtransferase
TFG: total free glutathione
TFC: total free cysteine
